# Mechanical Properties of Alginate Hydrogels Cross-Linked with Multivalent Cations

**DOI:** 10.3390/polym15143012

**Published:** 2023-07-12

**Authors:** Haniyeh Malektaj, Aleksey D. Drozdov, Jesper deClaville Christiansen

**Affiliations:** Department of Materials and Production, Aalborg University, Fibigerstraede 16, 9220 Aalborg, Denmark; aleksey@mp.aau.dk (A.D.D.); jc@mp.aau.dk (J.d.C.)

**Keywords:** alginate, hydrogel, multivalent cations, mechanical properties, equilibrium swelling

## Abstract

Ionically, cross-linked alginate gels have a potential to be used in a wide range of biomedical, environmental and catalytic applications. The study deals with preparation of alginate hydrogels cross-linked with various cations and the analysis of their equilibrium swelling and mechanical properties. It is shown that the type of cations used in the cross-linking process affects the elastic moduli and the equilibrium degree of swelling of the gels. The experimental data in small-amplitude oscillatory tests are fitted with a model that involves two material parameters: the elastic modulus of a polymer network and a measure of its inhomogeneity. The influence of cations on these quantities is studied numerically. It is revealed that the dependence of the elastic modulus of ionically cross-linked alginate gels on their equilibrium degree of swelling differs from that predicted by the conventional theory for covalently cross-linked gels.

## 1. Introduction

Alginate is a natural polysaccharide with excellent biodegradability and biocompatibility. Alginate-based hydrogels have found numerous applications in regenerative medicine [1,2,3], drug delivery [4,5], cell carriers [6], wound healing [7], tissue regeneration [8,9], as well as in environmental applications [10,11], flexible electronics [12,13], and food industry [14,15,16].

Alginate is composed of irregular blocks of β-d-mannuronic acid (M) and α-l-guluronic acid (G) residues [1]. CaCl_2_ is a commonly used salt to cross-link alginate hydrogels. However, it causes fast gelation that leads to heterogenous cross-linking of the gels [17]. Alginate hydrogels with slower gelation show better structural uniformity and a higher elastic modulus than rapidly formed hydrogels [18]. Calcium salts with low solubility in pure water (CaCO_3_ and CaSO_4_) can become soluble using chemical reactions, resulting in uniform distribution of Ca^2+^ ions and gradual gelation of alginate hydrogels. The slow gelation of alginate was reported by the in situ release of Ca^2+^ from CaCO_3_ and CaSO_4_ particles induced by hydrolysis of GDL (d-glucono-δ-lactone) to reduce pH [19,20], which led to the formation of gels with a uniform structure and superior mechanical properties as scaffold materials. Characterization of the mechanical properties of gels made with CaCO_3_-GDL showed that compressive modulus and strength increased with calcium content due to an increase in the cross-linking density [18].

Mechanical properties play a crucial role in determining the suitability of hydrogels for various applications. The type of cations that are used for cross-linking and the strength of interactions between ions and alginate chains influence significantly the mechanical properties of the ionically cross-linked alginate hydrogel. Aqueous solution of alginate can be cross-linked by various divalent and trivalent cations such as Ca^2+^, Sr^2+^, Ba^2+^, Cu^2+^, Zn^2+^, Mn^2+^, Fe^2+^, Cr^3+^, and Fe^3+^ [21,22,23,24]. Alginate hydrogels cross-linked with Ca^2+^ ions exhibit typically the elastic moduli in the range of 1–100 kPa [25,26]. Their values depend on the concentration of cations and the composition of alginate chains. The mechanical properties of alginate gels cross-linked by multivalent cations can be tuned by changing the type of cations [27]. The elastic moduli of Ca–alginate and Ba–alginate hydrogels prepared from 2 wt.% alginate solution in water with the same concentration of CaCl_2_ or BaCl_2_ cations (ranged from 0.5 to 10 wt.%) change in the intervals between 5 and 12 kPa, and between 7 and 20 kPa, respectively [28]. The degree of swelling can also be controlled by adjusting the concentration and type of multivalent cations used in the cross-linking process. Marcus et al. [29] showed that alginate microgels cross-linked with Ca^2+^ ions had a higher degree of swelling compared with those prepared with Ba^2+^ cations. The control over swelling behavior provides an opportunity to design hydrogels with tailored properties, such as targeted release of encapsulated cargos or responsiveness to environmental stimuli [30].

To widen applications of alginate hydrogels, some researchers combined Ca^2+^ with other metal ions such as Ba^2+^ and Al^3+^ [24,31]. An important opportunity in the preparation of alginate gels is to select and mix ions based on their biological effects. In [32], strontium was employed in combination with calcium to cross-link alginate chains in order to enhance the osteogenic differentiation. Alginate gels cross-linked with Zn^2+^ ions are highly toxic. An addition of calcium ions to zinc ions reduces toxicity of alginate gels. Alginate gels cross-linked with a mixture of zinc and calcium ions are efficient in protecting surfaces by blocking adhesion of microalgae [33]. Wang et al. [34] revealed that alginate fibers prepared by using Ca-Cu, Ca-Ba, and Ca-Zn ionic systems exhibited a substantial decrease in bacterial growth in comparison to alginate fibers cross-linked with Ca ions. Moreover, alginate fibers cross-linked with hybrid ions demonstrated superior mechanical strength and toughness compared with Ca–alginate fibers. Shaheen et al. [35] explored the effect of direct conjugation of two metal ions on the rheological and swelling properties of an alginate gel as compared to their single metal ion counterparts. It was observed that the bimetallic hydrogels usually possess superior self-healing ability, swelling properties, water retention capability, and mechanical strength as compared to the monometallic alginate hydrogels.

The mechanical properties of ionically cross-linked alginate hydrogels are changed when gels are exposed to physiological media. The hydrogels disintegrate in the presence of chelating agents, monovalent ions, and non-cross-linking divalent ions found in tissue culture medium and fluids [36,37]. It was also reported that alginate hydrogels cross-linked with various cations can be prepared by using seawater instead of pure water without losing their mechanical properties. This provides an advantage in large-scale hydrogel production by saving pure water [38]. 

In [21], a two-step cross-linking process was used to prepare an ionically cross-linked Ca–alginate gel. This method ensures uniform cross-linking of polymer chains, resulting in stable gels that can withstand degradation in a 0–0.2 M NaCl solution. A similar approach is used in the present work. At the first step, alginate chains are weakly cross-linked by Ca^2+^ in an aqueous solution with a low pH. At the next step, the gels are immersed into a strong solution of various salts to complete the cross-linking process.

The objective of this study is to investigate the mechanical properties and the equilibrium swelling of homogeneous alginate hydrogels cross-linked with various divalent and trivalent ions (Ca^2+^, Sr^2+^, Cu^2+^, Zn^2+^, and Fe^3+^). In previous works, mechanical and swelling properties were not analyzed simultaneously. Dynamic mechanical analysis (DMA) is used to characterize the mechanical properties of hydrogels. It was found that the trivalent cation (Fe^3+^) has a stronger effect on the mechanical properties than divalent cations. The mechanical properties of the hydrogels cross-linked with divalent ions are noticeably affected by the affinity of cations to alginate chains (the interactions between cations and M and G blocks of alginate chains). The hydrogels under investigation have potential applications in artificial tissues and structural materials.

## 2. Materials and Methods

### 2.1. Materials

Alginic acid sodium salt from brown algae was purchased from Acros Organics (Geel, Belgium). Calcium chloride (CaCl_2_) was provided by Merck, Burlington, MA, USA). Copper chloride (CuCl_2_), and iron III chloride hexahydrate (FeCl_3_·6H_2_O) were purchased from Sigma-Aldrich, St. Louis, MO, USA. Strontium chloride hexahydrate (SrCl_2_·6H_2_O) was provided by Strem Chemicals, Newburyport, MA, USA. Zinc chloride (ZnCl_2_) was provided by Honeywell, Seelze, Germany. Hydrochloric acid (HCl), 37% (*v*/*v*), was supplied by VWR International (Rosny-sous-Bois, France). Deionized water was used in preparation and testing of hydrogels.

### 2.2. Preparation of Hydrogels

Several series of ionically cross-linked alginate gels were prepared with different salts by a method described previously in [21]. First, pH of an alginate solution (1 wt.%) was reduced to 3.5 by the addition of HCl in order to reduce the ionization of carboxyl groups at the alginate backbone. The alginate solution was mixed with 50 mM of CaCl_2_ solution in proportion 28:1 (*v*/*v*). The mixture was poured into a mold and kept at room temperature overnight to prepare a weak gel. These weak hydrogels were immersed into a CaCl_2_ (1 M), CaCl_2_ (0.1 M), SrCl_2_ (1 M), CuCl_2_ (1 M), ZnCl_2_ (1 M), or FeCl_3_ (0.1 M) solutions for 2 days to complete the cross-linking process. Afterwards, all hydrogels were immersed into neutral water overnight to remove unreacted moieties.

### 2.3. Mechanical Tests

The DMA Q800 V20.9 (TA Instruments, New Castle, DE, USA) was used to measure the storage modulus $E^{\prime }$ and the loss modulus $E^{\prime \prime }$ in small-amplitude compressive oscillatory tests at temperature T = 22 °C with strain amplitude of 0.5% and frequency *f* ranged from 0.1 to 60 Hz. The measurements were conducted three times on disc-shape samples with a diameter of 8.5 mm and height of 4.5 mm. The standard deviations of the data did not exceed 5% of their mean values. 

### 2.4. Swelling Tests

Equilibrium swelling tests were conducted on alginate hydrogels immersed in 1 L aqueous solutions with pH = 7 at temperature T = 22 °C. Pre-weighted disc samples with diameter of 8.5 mm and height of 2.5 mm were immersed in aqueous solution with pH = 7, and their equilibrium weights were measured after three days. This duration exceeds the time (assessed in [21]) necessary for the samples to reach their equilibrium. The equilibrium degree of swelling $Q_{\infty }$ was determined by the formula:

(1) $$Q_{\infty }=\frac{w_{\infty }-w_{0}}{w_{0}}\;$$

where $w_{0}$ and $w_{\infty }$ stand for the weight of a dry sample and its weight under equilibrium, respectively.

The measurements were repeated five times on different disc-shape samples. The experimental data are presented as the mean values. The scatter of the data is relatively small, with the standard deviations that do not exceed 5% of the mean values.

## 3. Results

### 3.1. The Mechanical Properties of Alginate Hydrogels

**Figure 1:**
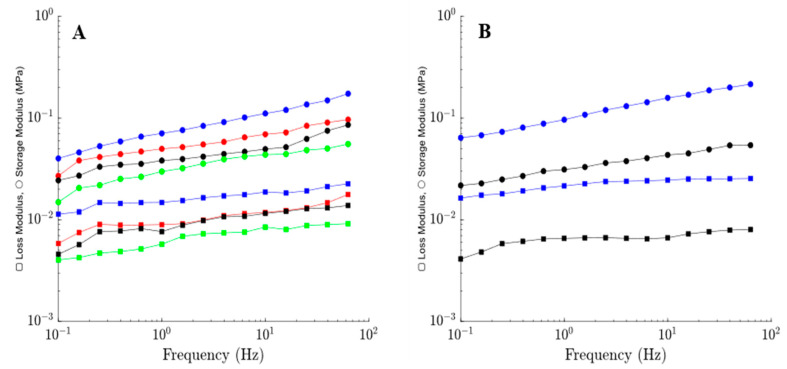
Storage modulus $E^{\prime }$ (circles) and loss modulus $E^{\prime \prime }$ (squares) versus frequency *f* for alginate hydrogels cross-linked with (**A**) 1 M of CuCl_2_ (blue), SrCl_2_ (red), CaCl_2_ (black), ZnCl_2_ (green), and (**B**) 0.1 M of FeCl_3_ (blue), CaCl_2_ (black).

To assess the effect of cations on the mechanical properties of alginate hydrogels, the storage modulus $E^{\prime }$ and loss modulus $E^{\prime \prime }$ were measured in small-amplitude oscillatory tests. The effect of frequency *f* on the storage $E^{\prime }$ and loss $E^{\prime \prime }$ moduli of gels prepared with 1 M divalent cations is illustrated in Figure 1A. Since the gels prepared with 1 M FeCl_3_ had very low stretchability and could not maintain their structural integrity (results not shown) [35], a lower concentration (0.1 M) of FeCl_3_ was chosen. At higher concentrations, aggregation of FeCl_3_ occurs, leading to the destruction of bonds [39]. For comparison, the storage $E^{\prime }$ and loss $E^{\prime \prime }$ moduli of Ca–alginate prepared with 0.1 M CaCl_2_ were presented in Figure 1B. The storage $E^{\prime }$ and loss $E^{\prime \prime }$ moduli of the gels prepared with various divalent cations decrease in the order Cu–alginate > Sr–alginate > Ca–alginate > Zn–alginate in the entire frequency *f* range between 0.1 and 60 Hz. Compared to divalent cations, Fe^3+^ ions induce a strong increase in the storage and loss moduli.

Each set of data in Figure 1A,B was fitted separately by using the model discussed in [40]. The storage $E^{\prime }(\omega )$ and loss $E^{\prime \prime }(\omega )$ moduli are given by

(2) $$E^{\prime }\left(\omega \right)=E\int_{0}^{\infty }f\left(v\right)\frac{\omega^{2}}{\Gamma^{2}\left(v\right)+\omega^{2}}dv$$

(3) $$E^{\prime \prime }\left(\omega \right)=E\int_{0}^{\infty }f\left(v\right)\frac{\Gamma \left(v\right)\omega }{\Gamma^{2}\left(v\right)+\omega^{2}}dv$$

The rate of rearrangement of reversible bonds between chains 𝛤(𝑣) is determined by the Eyring formula:

(4) $$\Gamma \left(v\right)=\Gamma_{0}\exp\;\left(-v\right)$$

where v is a dimensionless activation energy, and $\Gamma_{0}$ is a pre-factor, which is considered to be constant 15.000 s^−1^.

The inhomogeneity of a polymer network is characterized by the probability density f(v) to find a reversible bond with an activation energy v. The latter is described by the quasi-Gaussian formula:

(5) $$f\left(v\right)=f_{0}\exp\left(-\frac{v^{2}}{2\;\Sigma^{2}}\right)$$

where Σ is a measure of inhomogeneity. 

The model treats a gel as a temporary network of flexible polymer chains connected by reversible bonds. The bonds can break and reform at random instants. Rearrangement of transient bonds occurs due to thermal fluctuations. Each bond is characterized by the activation energy *v* necessary for its breakage. The distribution of bonds with various activation energies is determined by the function $f\left(v\right)$ given by Equation (5), where Σ characterizes the inhomogeneity of the network. When a temporary bond breaks, the stresses in chains connected by this bond vanish. When a new temporary bond is formed, the initial state of the chains merged by this bond coincides with the current state of the network. In accord with the “egg-box” model for ionically cross-linked alginate gels [41], the inhomogeneity of a polymer network is affected by the number of cations and G-blocks forming a particular zipping structure between two nearby chains, as well as the strength of ionic interactions between them.

**Figure 2:**
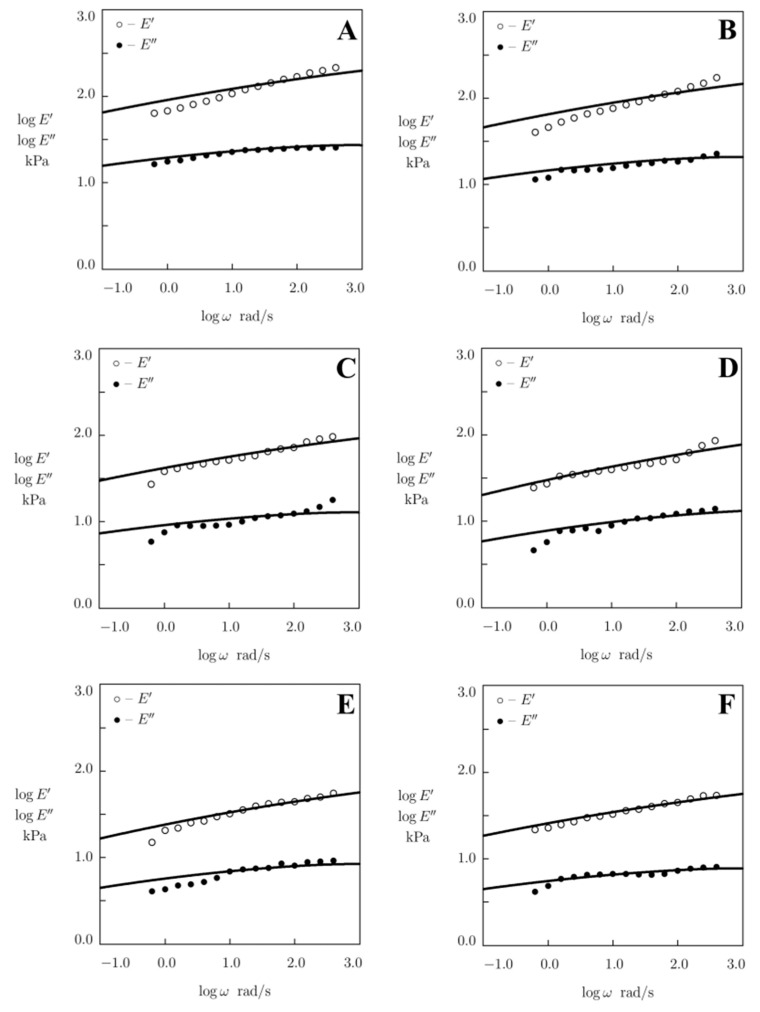
Storage modulus $E^{\prime }$ and loss modulus $E^{\prime \prime }$ versus angular frequency ω. Symbols: experimental data on alginate gels cross-linked with (**A**) Fe^3+^ ions (0.1 M), (**B**) Cu^2+^ ions (1 M), (**C**) Sr^2+^ ions (1 M), (**D**) Ca^2+^ ions (1 M), (**E**) Zn^2+^ ions (1 M), and (**F**) Ca^2+^ ions (0.1 M). Solid lines: results of numerical analysis.

Given an angular frequency ω=2πf, Equations (2) and (3) together with Equation (4) for $\Gamma \left(v\right)$ and Equation (5) for $f\left(v\right)$ involve two material parameters: (i) *E* stands for the elastic modulus of a gel, and (ii) Σ is a measure of inhomogeneity of the polymer network. These coefficients are found by fitting experimental data depicted in Figure 2. Each set of observations is matched separately by means of the nonlinear regression method. Figure 2 demonstrates an acceptable agreement between the experimental data and results of numerical simulation.

The values of *E* and Σ for all gels under consideration are presented in Table 1. The data show that the coefficient Σ is practically independent of the type of cations, and the difference between the maximum 10.6 and the minimum 9.9 values of Σ is around 6%. The elastic modulus *E* of the gel cross-linked with 0.1 M Fe^3+^ ions exceed that for the gels with 0.1 M Ca^2+^ ions by a factor of 3.5. The type of divalent cations cross-linking the polymer network affects the elastic modulus *E* of hydrogels. The modulus *E* decreases in the following order: Cu^2+^ > Sr^2+^ = Ca^2+^ > Zn^2+^. 

### 3.2. The Equilibrium Degree of Swelling of Alginate Hydrogels

**Figure 3:**
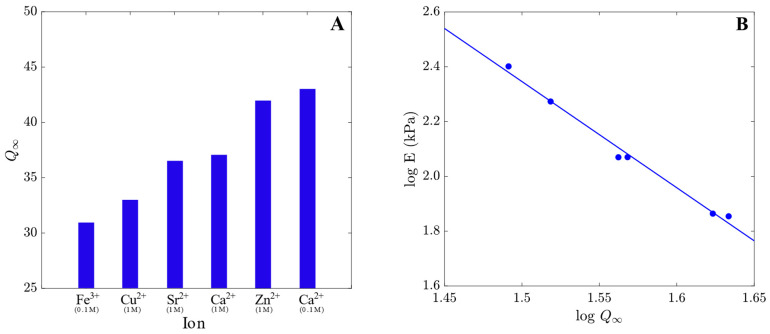
(**A**) Equilibrium degree of swelling $Q_{\infty }$ of alginate hydrogels cross-linked with various cations, and (**B**) The elastic modulus *E* versus equilibrium degree of swelling $Q_{\infty }$. Circles: experimental data. Solid line: their approximation by Equation (7).

To analyze the influence of cations on the equilibrium degree of swelling, samples of alginate gels were immersed in solutions with pH = 7, and their equilibrium degrees of swelling $Q_{\infty }$ were measured. The results are presented in Figure 3A where equilibrium degree of swelling $Q_{\infty }$ was shown for each cation. The equilibrium degree of swelling $Q_{\infty }$ is affected by the salt used in the preparation procedure, and it grows with a decrease in the elastic modulus *E*. 

The dependence of *E* on $Q_{\infty }$ was approximated by Equation [42],

(6) $$E\sim \frac{1}{Q_{\infty }^{B}},$$

which can be presented in the form

(7) $$\log E=A-B\;log{\;Q}_{\infty }.\;$$

The elastic modulus *E* is plotted in Figure 3B as a function of the equilibrium degree of swelling $Q_{\infty }$. The coefficient *B* = 3.9 was determined using the least-square method. This value differs from those predicted by the conventional theories for covalently cross-linked gels (according to these theories, *B* ranges from 1/3 to 2/3).

## 4. Discussion 

We prepared alginate hydrogels cross-linked with various multivalent cations (Fe^3+^, Cu^2+^, Sr^2+^, Ca^2+^, Zn^2+^) and investigated their mechanical properties by means of DMA. 

The experimental study involved small-amplitude oscillatory tests in a frequency *f* range from 0.1 to 60 Hz. It is revealed that the $E^{\prime \prime }$ and $E^{\prime }$ values are affected by cations. A model was used to approximate each set of $E^{\prime \prime }$ and $E^{\prime }$ data in the small-amplitude oscillatory tests. It is found that trivalent ions (Fe^3+^) have a stronger effect on the elastic modulus *E*, compared to divalent ions. The elastic modulus *E* of alginate gels cross-linked with divalent ions decreases in the following order: Cu^2+^ > Sr^2+^ = Ca^2+^ > Zn^2+^. The divalent ions form two-dimensional egg-box structures with alginate [43], while the trivalent cations are able to form three-dimensional structures [35,44]. Trivalent cations can interact with three carboxylic groups of different alginate chains at the same time. This leads to an increase in the coordination number and formation of a 3D bonding structure [35,44,45].

Molecular modeling and nuclear magnetic resonance (NMR) spectroscopy demonstrated that charge and ion radius of multivalent cations can affect interaction of ions with alginic acid, and the charge may play a more critical role [45,46]. A relationship between the mechanical properties and the ionic radius of divalent ions was introduced in [45], where it was shown that Ba^2+^ cations with a larger ion radius compared to Ca^2+^ can form a tighter structure than Ca^2+^. This is because Ba^2+^ cations are able to fill a larger space between the blocks of alginate polymers, resulting in a more tightly arranged structure [45]. However, this hypothesis is applicable mostly for cations of the same group. We showed that although Cu^2+^ has a smaller ionic radius than Sr^2+^, it has a higher elastic modulus. Therefore, factors other than ionic radius affect the mechanical properties of alginate gels.

The mechanical properties of alginate hydrogels depend on the interaction between cations and GG blocks, MM blocks, and MG blocks [45,47]. Fe^3+^ ions can combine with GG, MM, and MG blocks in alginate resulting in enhanced strength of the hydrogel [48]. For the trivalent lanthanide ions, there is a preference for GG blocks over MM blocks, while GG and MM blocks exhibit a stronger binding affinity as the charge density increases [46]. The affinity of alginate toward different divalent ions decreases in the following order:  Pb > Cu > Cd > Ba > Sr > Ca > Co= Ni= Zn > Mn [49,50,51]. This order agrees with our results and can be explained by the interaction between divalent cations and alginate blocks. According to the “egg-box” model, each Ca^2+^ ion is coordinated by four guluronate units [46], two from each chain. More coordination sites of Sr^2+^ with alginate molecule than Ca^2+^ were reported in [52], which resulted in a stronger binding of Sr^2+^ with alginate [52,53]. However, Sr^2+^ is likely to bind only to GG complex [27], whereas Ca^2+^ binds to both GG and MG blocks, while Zn^2+^ binds to all MM, GG, and MG blocks [54]. However, Zn^2+^ lacks specificity and binds randomly with GG, MM, and MG blocks. In addition, since Zn^2+^ has a low affinity for binding to alginate, it results in a looser network with a higher equilibrium degree of swelling and lower tensile properties [54]. Although Cu^2+^ ions are less selective for binding to alginate [55], their strong binding affinity led to the formation of a rigid structure [56,57]. The lowest release of Cu^2+^ from alginate films compared to Ca^2+^ and Zn^2+^ was reported in [55] and explained by the higher binding affinity of Cu^2+^. 

Moreover, coordination chemistry also plays an important role in the strength of the cation–alginate complexes [58,59]. In [60], the computed bond distances, cation interaction energies, and molecular orbital compositions revealed that the interaction between uronate units and alkaline earth metal ions is purely electrostatic, while transition metals establish strong covalent–coordination bonds. Alkaline earth metal ions have a relatively low charge density and larger ionic radius, making them more likely to form electrostatic interactions rather than strong covalent bonds with the carboxylate groups. The partially filled d-orbitals of copper facilitate additional interactions, such as d-orbital overlap with carboxylate groups. These additional interactions contribute to the formation of covalent coordination bonds, which enhance the strength of the Cu–alginate complexes. On the other hand, Zn–alginate primarily involves electrostatic interactions. Although zinc can form coordination bonds, the absence of partially filled d-orbitals and a smaller ionic radius result in weaker covalent interactions compared to Cu^2+^.

The differences in the strength of cations–alginate gels can be attributed to a combination of factors, including the charge density, ionic radius, coordination chemistry, selectivity, and affinity of ions for binding to alginate and the interaction between cations and GG blocks, MM blocks, and MG blocks.

The equilibrium degree of swelling of alginate gels cross-linked with multivalent ions increases in the following order: Fe^3+^ < Cu^2+^ < Sr^2+^ < Ca^2+^ < Zn^2+^. The degree of swelling of Ca–alginate hydrogels increases with a decrease in the CaCl_2_ concentration in the cross-linking solution as reported in [37]. For practical use, high water content hydrogels should not only show good conductivity but should also be sufficiently strong and have good elastic properties [61]. The mechanical properties of hydrogels are greatly influenced by the fraction of water inside them which is characterized by the equilibrium degree of swelling $Q_{\infty }$ [61,62]. The elastic modulus *E* decreases with equilibrium degree of swelling $Q_{\infty }$. A similar behavior for alginate hydrogels was observed in [63]. Comparison of hydrogels prepared with 0.1 and 1 M CaCl_2_ in Figure 3 shows that at higher concentrations of Ca^2+^ ions, longer GG/GG junctions are formed that are more resistant to elastic deformation and have a higher elastic modulus *E*. 

Alginate gels cross-linked with various ions can be used in such applications as drug delivery, tissues engineering, skin grafting, and biocatalysts. There is an opportunity to select ions based on their known biological effects. Sr^2+^ or Cu^2+^ ions cannot be used to immobilize cells due to their potential toxic effect on cells [64,65], while the Ca^2+^ and Zn^2+^ cations can be used in drug immobilization [65]. Elements closely chemically related to Ca, such as Sr, have pharmacological and metabolic effects on bones in vivo. 

For example, Sr^2+^ is beneficial for the bone growth and repair through the activation of osteoblast activity and suppression of osteoclast (bone resorbing) function [32,66]. However, both positive and negative effects of Sr^2+^ on bone growth depend on the concentration of Sr^2+^ ions, availability of calcium in the diet, kidney function, and the animal model [67]. Studies show that Sr (as chloride) is not toxic for bone cells at low doses (lower than 1% or 4 mmol Sr/kg/day) [68,69], whereas higher doses can cause skeletal abnormalities, especially in animals with low-calcium diets [67]. Therefore, it is recommended to use Sr simultaneously with Ca for bone repair. 

Fe–alginate can overcome the deficiencies of Ca–alginate, such as poor protein adsorptive capacity. Fe–alginate gels can be used as an effective cell culture substrate [48]. The rich redox chemistry of Fe^3+^ cations can be exploited for a wide range of applications, such as drug delivery, tissue engineering, or environmental remediation [70]. Alginate hydrogels are characterized by a broad chemical modification capacity, which makes them suitable for applications as metal catalysts [71] and biocatalysts [72,73]. 

## 5. Conclusions

Alginate gels cross-linked with Fe^3+^, Cu^2+^, Sr^2+^, Ca^2+^, and Zn^2+^ ions have been prepared, and their mechanical and swelling properties have been studied. Each set of $E^{\prime }$ and $E^{\prime \prime }$ data in small-amplitude compressive oscillatory tests was approximated by a model with two parameters. An acceptable agreement is demonstrated between the data and results of simulation. Cross-linking with Fe^3+^ cations leads to a strong increase in the elastic modulus. The elastic moduli of alginate gels cross-linked with divalent ions decay in the order of Cu–alginate > Sr–alginate = Ca–alginate > Zn–alginate. The divalent ions form a 2D egg-box structure with alginate chains, while the binding extent of trivalent cations with alginate produces a more compact network. In addition to the charge density, the mechanical properties depend on the interaction between cations and GG blocks, MM blocks, and MG blocks of alginate. The elastic modulus *E* and the equilibrium degree of swelling $Q_{\infty }$ of ionically cross-linked alginate gels are connected by Equation (7) with a coefficient *B* that differs from those predicted by the conventional theories for covalently cross-linked hydrogels. 

## Figures and Tables

**Table 1:** Material parameters for alginate hydrogels cross-linked with various cations.

Ion	Σ	E (kPa)
Fe^3+^ (0.1 M)	10.6	252.0
Cu^2+^ (1 M)	10.3	187.6
Sr^2+^ (1 M)	10.5	117.4
Ca^2+^ (1 M)	9.9	117.5
Zn^2+^ (1 M)	9.9	73.1
Ca^2+^ (0.1 M)	10.6	71.5

## Data Availability

The data that support the findings of this study are available from the corresponding author, H.M., upon reasonable request.
